# Pathology and VP2-Based Characterization of Infectious Bursal Disease Virus Associated with an Outbreak in Layer Chickens in Ghana

**DOI:** 10.3390/pathogens13121115

**Published:** 2024-12-17

**Authors:** Ben Enyetornye, Henry A. Abugri, Ama K. Kusi-Appiah, Grazieli Maboni, Theophilus Odoom, Nicole L. Gottdenker, Binu T. Velayudhan

**Affiliations:** 1 Athens Veterinary Diagnostic Laboratory, College of Veterinary Medicine, University of Georgia, Athens, GA 30602, USA; be96354@uga.edu (B.E.); grazim@uga.edu (G.M.); 2 Department of Pathology, College of Veterinary Medicine, University of Georgia, Athens, GA 30602, USA; 3 School of Veterinary Medicine, University of Ghana, Legon, Accra P.O. Box LG139, Ghana; henryabugri@gmail.com (H.A.A.); keziah158@gmail.com (A.K.K.-A.); 4 Accra Veterinary Laboratory, Veterinary Services Directorate, Accra P.O. Box GA184, Ghana

**Keywords:** chickens, Ghana, infectious bursal disease, pathology, phylogenetic analysis

## Abstract

Infectious bursal disease (IBD) continues to threaten poultry production globally, with highly virulent strains circulating in many parts of Africa. In this study, molecular characterization was performed on a circulating infectious bursal disease virus (IBDV) strain from an outbreak in a layer flock in Ghana. Layer chicks presented for necropsy had markedly enlarged and hemorrhagic bursae of Fabricius, with necrotic foci and catarrhal exudate on the serosal surface. Histopathology of the bursa of Fabricius revealed scattered to effacing hemorrhages on the plicae, extensive necrosis with expansion of the stroma between the follicles, and depletion of lymphocytes within the interfollicular epithelium. Reverse transcription polymerase chain reaction (RT-PCR) and subsequent sequencing of the VP2 gene showed the presence of IBDV in formalin-fixed paraffin-embedded tissues. A phylogenetic analysis compared 62 other IBDV sequences from different parts of the world and placed the Ghanaian IBDV in genogroup 3 (vvIBDV), closely related to IBDV from Nigeria. In comparison to reference vvIBDV, there were amino acid substitutions at positions 252, 254, and 300. To the best of our knowledge, this is the first report in which an IBDV from a disease outbreak in Ghana has been sequenced and compared with other IBDVs in a phylogenetic analysis.

## 1. Introduction

Infectious bursal disease or Gumboro disease is one of the most detrimental and economically important immunosuppressive diseases of growing chickens, with high prevalence worldwide [1,2]. Over the past 66 years, infectious bursal disease has caused significant morbidity and mortality globally, especially in most African nations where the disease is endemic [3]. In Saskatchewan in Canada, the poultry industry loses 3.9 million kilograms of meat production per year due to variant IBDV strains [4]. The etiological agent, infectious bursal disease virus (IBDV), primarily infects actively dividing pre-B-lymphocytes in the bursa of Fabricius [5,6], thereby suppressing the immune system of affected birds [7] and predisposing them to other viral and secondary bacterial infections [8]. This disease hinders poultry production in many parts of the world [9], especially in African countries like Ghana, where poultry is a major livelihood source, particularly for rural dwellers [10]. In some African countries like Nigeria, Senegal, Sudan, and Tanzania, very virulent infectious bursa disease virus (vvIBDV) strains have been reported to be circulating in the field [11,12,13,14].

In Ghana, IBD was first reported in 1976, and for many years following that, the virus was recognized in a mild form, accounting for about 2 to 5% of poultry mortality [15]. Reports from the Veterinary Services Directorate of Ghana in 2001 suggested that the mild virus strain evolved into a very virulent strain, contributing to about 25 and 60% mortality of broiler and layer flocks, respectively [16]. Further characterization of field strains by reverse transcription polymerase chain reaction–restriction fragment length polymorphism (RT-PCR-RFLP) suggests that the field isolates circulating in Ghana were highly virulent [16]. In 2009, an epidemiological study indicated that IBD was a major challenge for poultry producers, with many cases recorded in the regions of Accra and Kumasi, Ghana [17].

To date, no molecular characterization (sequencing) of the circulating IBDV in Ghana has been performed. IBDV is classified into two serotypes (serotypes 1 and 2) based on antigenicity, with serotype 1 being pathogenic in chickens [18,19]. Serotype 1 viruses are further categorized based on pathogenicity into classical IBDV (cIBDV), variant IBDV (varIBDV), very virulent IBDV (vvIBDV), attenuated IBDV, and novel variant IBDV (nVarIBDV) [20,21]. Based on phylogenetic analysis of the hvVP2 of IBDV strains globally, serotype 1 IBDV has been classified into seven main genogroups (antigenic variant, vvIBDV, classical, ITA, Variant/classical recombinant, dIBDV, and Australian) [22]. Some studies also characterize IBDV using the hypervariable region of VP2 focusing on unique markers in comparison with well-characterized IBDV globally [23]. Other studies have also proposed IBDV classification based on sequence analysis of both hvVP2 and VP1 genes [24,25,26].

Here, we describe the pathological features of an outbreak in a White Leghorn (WLH) layer flock in Ghana in the year 2022. We also present a molecular characterization and phylogenetic comparative analysis of the IBDV associated with this outbreak in Ghana and IBDV viral sequences reported in various parts of the world based on the VP2 hypervariable region. To the best of our knowledge, this is the first documented report in which an IBDV detected in Ghana has been sequenced and used in phylogenetic comparative analysis to establish its relatedness to other IBD viruses from other countries. Although a limitation of our study may be that our characterization is solely based on the VP2 gene, this study sets the stage for further characterization of IBDVs circulating in various poultry production zones of Ghana.

## 2. Materials and Methods

### 2.1. Case Description

Eight four-week-old WLH chicks were submitted to the Accra Veterinary Laboratory, Ghana, for post-mortem evaluation in 2022. A day before the presentation, 10 birds were dull and completely off feed in the morning and died a few hours later. The next day of presentation for necropsy, mortality was 112 out of a total stock of 3000 birds. That is a mortality rate of 0.3% on the first day, increasing astronomically to 3.7% the next day with morbidity estimated to be around 60% (1800 birds). Further history suggested that the chicks were vaccinated against IBDV (at weeks 1 and 3 of age) and first Newcastle disease (at week two of age) as per Ghana’s national poultry vaccination schedule. The IBDV vaccine used was Nobilis Gumboro 228E strain, and Hitchner B1 strain was used for Newcastle disease vaccination. The chicks were fed commercially purchased layer feed.

### 2.2. Post-Mortem Evaluation

A thorough post-mortem examination was conducted, and gross images of lesions were captured and recorded. Representative tissue samples (bursa of Fabricius, spleen, and kidneys) were collected and kept in 10% buffered formalin with a pH of 7 at room temperature for 2 weeks before processing for histopathological evaluation.

### 2.3. RNA Extraction from Formalin-Fixed Paraffin-Embedded (FFPE) Tissues and RT-PCR

The FFPE tissue samples from four chicks were pooled based on organs (bursa of Fabricius, spleen, and kidney). Ten-micrometer sections of formalin-fixed, paraffin-embedded samples were taken from each tissue block, and about five pieces of each tissue section were used. RNA was extracted from all the three organ-pooled FFPE samples using Qiagen’s RNAeasy FFPE Kit (QIAGEN, Hilden, Germany) following the manufacturer’s instructions. Primers 743-F (5′-GCC CAGAGT CTACAC CAT-3′) and 1331-R (5′-ATG GCT CCT GGG TCA AAT CG-3′) were used to amplify a 579 bp fragment of the hypervariable region of the VP2 gene (hvVP2). RT-PCR was performed at 48 °C for 30 min and terminated by incubation at 95 °C for 10 min, and this was followed by 35 cycles of PCR at 95 °C for 30 s, 57 °C for 1.5 min and 72 °C for 1.5 min [22,27]. The VP2 gene was amplified by RT-PCR using Invitrogen’s SuperScript III One-Step RT-PCR System with Platinum Taq DNA Polymerase, and purification was performed using Qiagen’s QIAquick PCR Purification Kit (QIAGEN, Hilden, Germany) following the manufacturer’s instructions at the Poultry Diagnostic and Research Center at the University of Georgia, USA. The purified PCR product was sent to Eurofins Genomics, USA, for fragment gene sequencing based on the Sanger method.

### 2.4. Viruses Used in Phylogenetic Analysis

Based on previous studies that characterized IBDV [22,28], we obtained the accession numbers of IBDV viruses with known background from these studies and downloaded the viral sequences from GenBank. We also performed an NCBI blast of our IBDV sequence (accession number: PQ639438) and downloaded some sequences based on our blast results. We obtained a total of 62 IBDVs from GenBank with diverse backgrounds and used these viruses in addition to PQ639438 for phylogenetic analysis. IBDV with acession number AY321953 (F52-70 strain) was used as reference strain for classical IBDV (genogroup 1), AF133904 (DelE strain) and AF281238 (T1 strain) were used as reference for IBDV antigenic variants (genogroup 2), GQ221682 (rA strain) and AJ878898 (UK661 strain) were used as reference strains for vvIBDV (genogroup 3), and for dIBDV, JN982252 (MG4 strain), representing genogroup 4, was used. Mexico04M101 strain (DQ916210) and ITA-02 strain (JN852986) served as reference viruses for variant/classical recombinants (genogroup 5) and ITA (genogroup 6), respectively. The IBDV V877-W strain (HM071991) was used as reference for the Australian genogroup [22].

### 2.5. Phylogenetic Analysis

The phylogenetic tree was generated using the Maximum-Likelihood Method [29] based on the hypervariable domain of the VP2 capsid gene of IBDV using nucleotide alignment created in Clustal W, MEGA 11. This analysis involved 63 nucleotide sequences in total, which were retrieved from GenBank based on the published literature. All ambiguous positions were removed for each sequence pair (pairwise deletion option). Evolutionary analyses were conducted in MEGA11 with up to 1000 bootstrapping replicates [30]. The Newick file was then deposited in interactive tree of life (ITOL) and edited [31].

## 3. Results

### 3.1. Post-Mortem Findings

**Figure 1:**
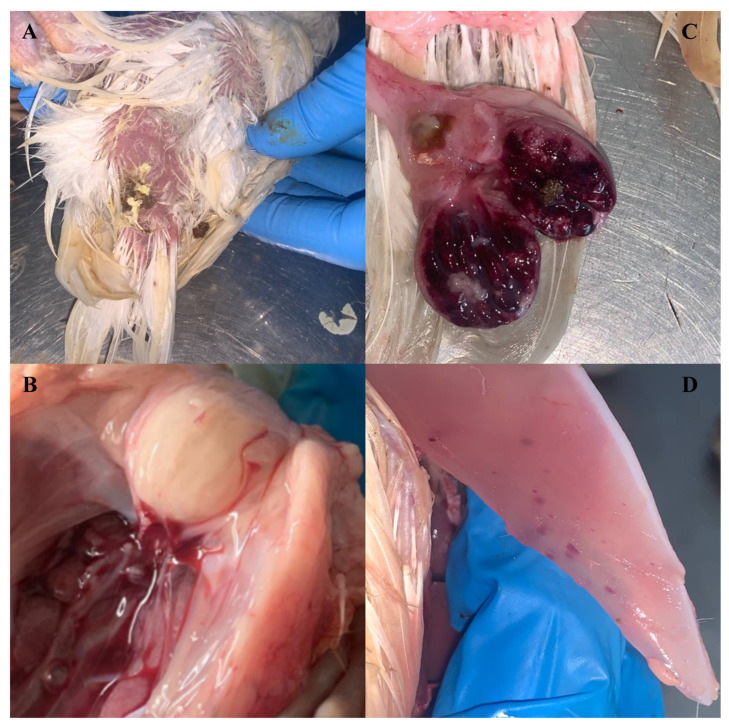
Gross necropsy lesions in layer chicks. (**A**) Vent soiled with white viscous to greenish fecal material (pasty vent), (**B**) markedly enlarged unopened bursa of Fabricius, (**C**) opened bursa of Fabricius which is hemorrhagic with necrotic foci and catarrhal exudate on the serosal surface, and (**D**) petechial to ecchymotic hemorrhage on the breast muscle.

Physical examination revealed dehydration, and in some birds, loose fecal material at the vent, indicating diarrhea. There were petechial hemorrhages on the pericardial fat (6/8). Multifocally, there were petechial to ecchymotic hemorrhages in the breast muscles (6/8). Paintbrush hemorrhages were evident on the thigh muscle (2/8). The bursa of Fabricius was enlarged three to four times compared to normal size in all eight birds (8/8). The cut surface of the bursa of Fabricius was hemorrhagic with necrotic foci and catarrhal exudate on the serosal surface (Figure 1).

### 3.2. Histopathology

**Figure 2:**
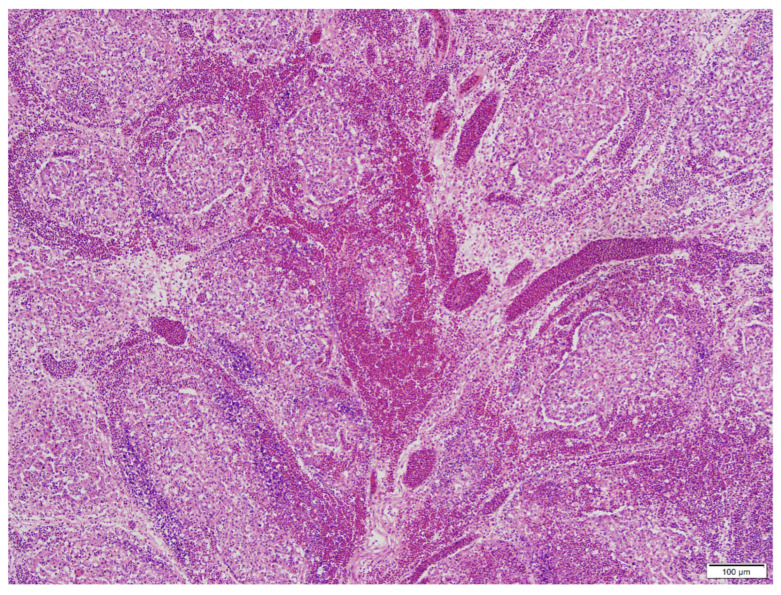
Extensive necro-hemorrhagic bursitis with expansion of the stroma between bursa follicles with edema fluid.

There were scattered to effacing hemorrhages on the plicae of the bursa of Fabricius. All the individual follicles were affected. Cells within affected follicles were highly eosinophilic with indistinct borders, abundant debris, and nuclei. Epithelium was multifocally and extensively absent and subtended by large spreading clusters of dense aggregates of lymphocytes. In many follicles, the outer follicular areas were extensively necrotic, with rare normal areas remaining. The stroma between the follicles was markedly expanded by edema. Intrafollicular epithelium was prominent in most follicles due to extensive loss of lymphocytes from necrosis (Figure 2).

Kidneys were severely congested with occasional small clusters of proximal tubular cells characterized by highly eosinophilic cytoplasm and karyorrhectic nuclei. Within the white pulp of the spleen, there was severe necrosis characterized by karyorrhectic and pyknotic nuclei and poor differentiation of cells. Within the red pulp, there was extensive vacuolar change with abundant macrophages and heterophils, indicating acute necrosis, characterized by indistinct cytoplasmic borders and karyorrhectic nuclei.

### 3.3. RT-PCR and Sequencing Results

All the pooled FFPE tissue samples (n = 3) tested positive for IBDV by RT-PCR. The amino acid sequence of the IBDV VP2 hypervariable region was 97.4% similar to vvIBDV. Amino acids consistent with vvIBDV were observed at positions 222 (Ala), 242 (Ile), 256 (Ile), 294 (Ile), and 299 (Ser) in our viral sequence (accession number: PQ639438). In comparison to reference vvIBDV strains (AJ878898 (UK661) and GQ221682 (rA)), amino acid substitution was observed at positions 252 (V→I), 254 (G→S), and 300 (E→A). The sequence of the IBDV vaccine used (accession number: AF457104, Nobilis Gumboro 228E) in comparison with the reference viruses and the field viral sequence (accession number: PQ639438) showed significant variation in deduced amino acid profile.

### 3.4. Phylogenetic Analysis

**Figure 3:**
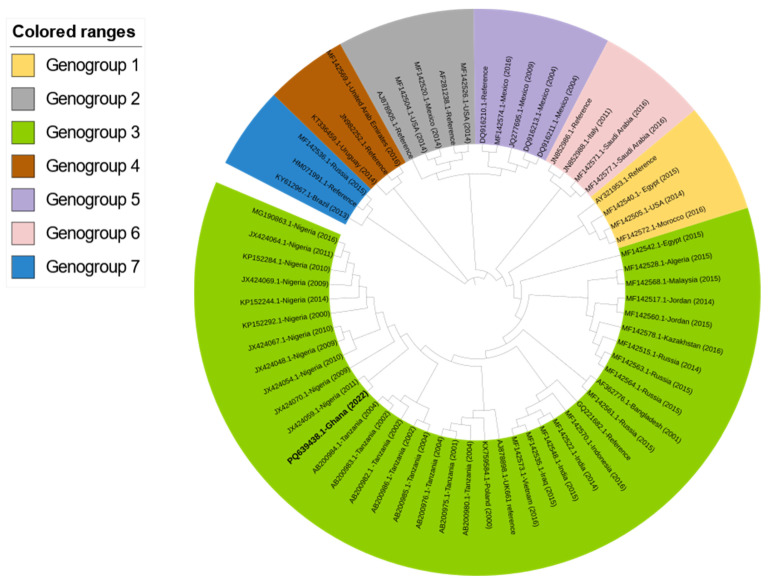
Phylogenetic tree was generated using the Maximum-Likelihood Method based on the hypervariable domain of the VP2 capsid gene of IBDV using nucleotide alignment created in Clustal W, MEGA 11. The Newick file was then deposited in an interactive tree of life (ITOL) and edited. The IBDV sequence with accession number PQ639438 (in bold) represents the IBDV reported in this study.

Phylogenetic comparative analysis placed the Ghanaian IBDV in genogroup 3 (vvIBDV), clustering with a Nigerian IBDV (accession number JX424059; Figure 3).

## 4. Discussion

This IBD outbreak in a layer farm in Ghana was confirmed by a combination of history, gross necropsy findings, histopathology, RT-PCR, and sequencing of the VP2 hypervariable gene. These methods are standard diagnostic techniques for IBD based on recommendations by the World Organization for Animal Health (WOAH, 2024). To the best of our knowledge, this is the first documented report in which an IBDV from Ghana has been sequenced and used in phylogenetic comparative analysis to establish its relatedness to other IBD viruses from other countries. To date, there are three documented studies on IBD in Ghana, which were published in the years 1976, 2008, and 2009 [15,16,17]. In 1976, the first outbreak of IBD in Ghana was reported, and in 2008, specific antibody-negative chickens were inoculated with IBDV isolates from various regions of Ghana in order to biologically pathotype (later confirmed by RT-PCR) the circulating IBDV strains. The most recent study in 2009 described the epidemiology of the virus, focusing on seasonality and distribution [15,16,17]. None of these studies sequenced the circulating strains of IBDV.

**Figure 4:**
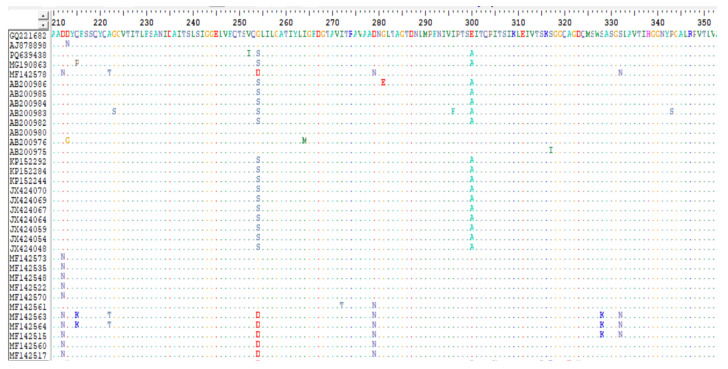
BioEdit analysis of deduced amino acid sequences of hvVP2 gene (210–350 aa) with rA and UK661 strains as reference strains. The alignment revealed the presence of vvIBD amino acid signatures in the IBDV (accession number: PQ639438) sequenced form an outbreak in layer chicks in Ghana.

**Figure 5:**
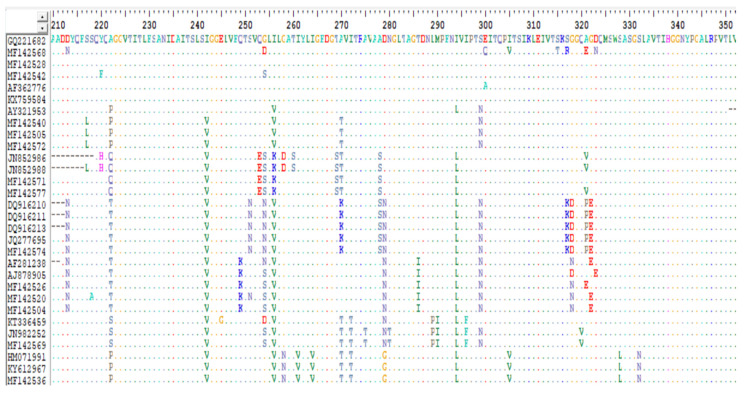
Continuation of BioEdit analysis of deduced amino acid sequences of hvVP2 gene (210–350 aa) with rA andUK661 strains as reference strains.

Examination of the VP2 hypervariable region of the IBDV reported in this study (accession number: PQ639438) revealed 97.4% similarity to the very virulent IBDV (vvIBDV) reference strains GQ221682 (rA strain) and AJ878898 (UK661 strain). This Ghanaian IBDV strain contained putative virulence marker amino acids observed at positions 222 (Ala), 242 (Ile), 256 (Ile), 294 (Ile), and 299 (Ser) (Figure 4 and Figure 5), which have been identified in most vvIBDV strains [28,32,33]. These virulent amino acid markers were reported to be conserved in vvIBDV isolated in Nigeria [34]. We also observed amino acid substitutions at positions 252 (V→I), 254 (G→S), and 300 (E→A) (Figure 4 and Figure 5). Substitutions of amino acids at position 300 (E→A) have been reported in 81 vvIBDV viruses isolated in Nigeria [34]. These observations emphasize the genetic relatedness of the Ghanaian IBDV to vvIBDV viruses in Nigeria.

These findings imply that the IBDV responsible for the outbreak in this layer flock was a very virulent strain, confirming studies by [16] where they pathotyped circulating IBDV in some parts of Ghana, but sequencing was not performed for further genomic comparison. In East Africa, a study conducted in Tanzania suggested that 14 circulating IBDVs reported from 2001 to 2004 were very virulent strains which, when analyzed phylogenetically, diverged into two genotypes, with one genotype closely related to IBD viruses from West Africa, while the other genotype clustered within the European/Asian very virulent type [28]. Another study that examined samples collected from 1997 to 2005 in 18 countries across four continents suggested that out of 113 samples, two IBDV isolates from South Africa were genetically similar to U.S. variant IBD viruses [35]. These observations imply that various strains of IBD viruses may be circulating on the African continent.

Generally, the common risk factors for IBD are the age of the birds, breed, immune status of the birds, viral virulence, farm biosecurity, and presence of secondary infections [36]. Some studies also specified breach in biosecurity, poor vaccine storage, use of few drinkers to administer vaccine, traces of disinfectants in drinkers used to administer live vaccines, use of wrong vaccine, and use of inappropriate diluents as IBD risk factors [37,38]. Although infectious bursal disease normally occurs in chicks between the ages of 3 and 6 weeks [36], it has been reported in chickens between 14 and 20 weeks, suggesting that age may not be a major barrier to disease occurrence as long as the birds are susceptible [39,40]. In this study, an IBD outbreak occurred in a 4-week-old White Leghorn (WLH) layer flock, an age which falls within the age range commonly reported for IBD outbreaks in chicken flocks. Generally, lighter breeds of chickens such as the WLH are considered more susceptible than heavier breeds [41]. Additionally, layer-type chickens of all genetic backgrounds show higher IBD viral antigenic loads in the bursa compared to the broiler breeds [42]. Perhaps this outbreak could be linked to the genetic susceptibility of WLH layer flocks to IBD, as suggested in previous studies [41]. A common practice in Ghana is to clean drinkers and feeders with detergent or sometimes disinfectants before rinsing with water. While the goal is to ensure these feeders and drinkers are devoid of any pathogen that the birds may be exposed to, disinfectants and detergent residue have been reported to potentially interfere with live vaccines, leading to vaccine failures [38]. On many poultry farms in Ghana, vaccines are diluted with tap water, well water, or water from bore holes. Since tap-borne water, which is commonly used, has been reported to contain chlorine due to treatment at the plant [43], farmers are normally advised to leave tap water overnight to allow for evaporation of chlorine, which could interfere with vaccines. In situations like this, chlorinated water can be neutralized by adding sodium thiosulphate (about 16 mg/L) [44], a practice which, if adopted, may be helpful to the farmers.

While IBDV antibodies have been detected [45] and the virus isolated [46,47,48] in wild birds, which are believed to play a role in the epidemiology of the disease, we suspect that this IBDV was introduced onto this layer farm either via fomites or farm workers since this layer flock was raised in a deep litter system with no access to wild birds. The chicks were fed on commercially purchased layer feed and trucks transporting feed to the farm could be a source of virus introduction if adequate biosecurity measures were not in place. Although the farmer did not report any previous outbreaks of IBD on the farm, IBDV has been reported to be very resistant to inactivation, accounting for virus persistence on poultry farms despite disinfection [49]. Hence, heightened biosecurity is important in its prevention and control. The IBDV was able to spread quickly in this flock, probably due to the deep litter management system used, aligning with reports in Nigeria, where an IBD outbreak has been reported in chicken raised in deep litter management systems [40]. In the deep litter management system (an intensive system of poultry keeping), there is free contact of the infected and non-infected chicks with chicks also having a direct access to their droppings, with high chances of feed and water contaminated by the droppings of infected chicks [50]. Perhaps this accounted for the 60% morbidity reported in this study, with associated 0.3% and 3.7% mortality rates on the first and second days of the onset of clinical signs of IBD. In Ethiopia, higher seroprevalence of IBD was recorded in birds raised in an intensive system compared with those kept in an extensive system [51], buttressing the point that chicks in close contact are at a higher risk. In fully susceptible flocks, the disease appears suddenly with a morbidity rate usually approaching 100%. Mortality may be nil but can be as high as 20–30% and exceptionally higher with vvIBDV [19]. In this study, the mortality is relatively low, probably because the two IBDV vaccines administered offered some partial protection against the disease.

In our study, the layer chicks received two doses of IBDV Nobilis Gumboro 228E vaccine at 1 and 3 weeks of age following the prescribed national vaccination protocol by the Veterinary Services Directorate of Ghana. Elsewhere, reports suggest that IBD outbreaks continue to be a challenge despite routine vaccination. Antigenic variations in the IBDV genome may be the cause of vaccine failure [52]. Infectious bursal disease outbreak was reported in a 14-week-old layer flock which received two doses of IBDV vaccine just like the chicks in this study, pointing to possible vaccine failure [40]. A study conducted in Tanzania, an East African country, suggests that the field strains of circulating IBDV were genetically different from strain in vaccines used to vaccinate birds against the disease, making vaccinated birds still succumb to the disease [14]. In our case, potential causes of this outbreak could be linked to improper vaccination, a break in the vaccine cold chain, antigenic variation in circulating IBDV, and possible neutralization of vaccine by high maternal antibodies passed on to the chicks [14,38,53]. Antibody levels of chicks prior to vaccination were not measured; hence, this cannot be ascertained, especially because measuring antibody levels prior to vaccination is uncommon in Ghana. In any case, some vaccines may not induce full protection against very virulent IBDV and antigenic variants reported in the last three decades [54]. Although the situation is gradually changing across the African landscape, it is quite difficult to maintain the vaccine cold chain due to the erratic power supply [40].

**Figure 6:**
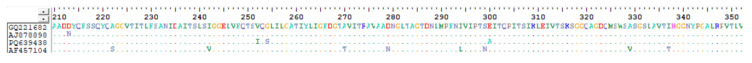
BioEdit analysis of deduced amino acid sequences of hvVP2 gene (210–350 aa) with rA and UK661 strains as reference strains. This alignment incorporated the IBDV vaccine strain (accession number: AF457104, Nobilis Gumboro 228E) used in vaccinating the birds. The alignment shows vast amino acid differences between the reference strain and the vaccine strain.

The amino acid profile comparison of this Ghanaian IBDV (accession number: PQ639438) with the vaccine strain used for vaccination suggests that the cause of this outbreak is not related to the vaccine strain but rather to a field IBDV since there was vast disparity in the deduced amino acid profiles of these two viruses (Figure 6). A VP2 sequence of the IBDV from this outbreak was used in conjunction with 62 other IBDV sequences retrieved from GenBank for phylogenetic analysis. The Ghanaian IBDV sequence was in a clade with other vvIBDVs belonging to genotype 3 and clustering with a vvIBD (JX424070-Nigeria (2009)) reported in Nigeria. This finding could be linked to the porous nature of the borders in many African countries, allowing the free flow of potentially infected live poultry and/or contaminated poultry products from neighboring countries, creating an avenue for pathogen spread [55,56]. Our findings align with the studies conducted in Namibia, where they observed that the circulating Namibian IBDV was genetically similar to IBDV circulating in Zambia, a neighboring African country.

It is, however, possible that this Ghanaian IBDV (PQ639438) was probably similar to other IBDVs in some other African countries but underwent viral evolution over time, making it relatively distinct. Infectious bursal disease viral evolution leading to the emergence of new or novel IBD viruses has been described in other studies [57]. Our results, therefore, warrant a more comprehensive study applying advanced molecular techniques, focusing on both VP2 and VP1 genes, to characterize circulating IBDV strains in all 16 regions of Ghana to enhance our understanding of the epidemiology and evolution of IBDV in Ghana. This will enable us to appreciate the burden of the disease as IBDV prevalence in many African countries, including Ghana, remains unknown. The newly detected IBD viruses would have to be matched with all vaccine strains available for use in Ghana to inform the proper implementation of preventive and control measures.

## 5. Conclusions

This study confirms that the cause of high morbidity and mortality in this group of layer chicks was a very virulent IBDV, which was genetically similar to other virulent IBDVs (genotype 3 IBDV) based on phylogenetic comparative analysis, clustering with a Nigerian IBDV isolate (GenBank accession number JX424070). We have, for the first time, sequenced, characterized, and described the pathology associated with IBDV in a White Leghorn layer flock raised in a deep litter system in Ghana. This IBDV (GenBank accession number: PQ639438) accounted for 0.3% and 3.7% mortality on the first and second days, respectively, when clinical signs began to manifest. These mortality rates were reported despite two IBDV vaccinations. In comparison to reference vvIBDV strains (AJ878898 (UK661) and GQ221682 (rA)), we observed amino acid substitution at positions 252 (V→I), 254 (G→S), and 300 (E→A). We also identified a significant variation in the amino acid profile of this Ghanaian IBDV sequence in comparison with the vaccine strain used in vaccinating the chicks, suggesting that the IBDV responsible for this outbreak is a field virus.

## Data Availability

The original contributions presented in the study are included in the article; further inquiries can be directed to the corresponding authors.

## References

[1] Thomrongsuwannakij T., Charoenvisal N., Chansiripornchai N. (2021). Comparison of two attenuated infectious bursal disease vaccine strains focused on safety and antibody response in commercial broilers. Vet. World.

[2] Mossie T. (2021). A Comprehensive Review on Infectious Bursal Disease Which Has Serious Threat for Ethiopian Poultry Sector. J. Biol. Agric. Healthc..

[3] Du X., Ahmad L., Wang B., Ding M., Elsaid F.G., Wen H., Yang J., Khan A. (2023). Infectious Bursal Disease: Distribution, Pathogenesis and Pathology. Pak. Vet. J..

[4] Zachar T., Popowich S., Goodhope B., Knezacek T., Ojkic D., Willson P., Ahmed K.A., Gomis S. (2016). A 5-year study of the incidence and economic impact of variant infectious bursal disease viruses on broiler production in Saskatchewan, Canada. Can. J. Vet. Res..

[5] Mahgoub H.A. (2012). An overview of infectious bursal disease. Arch. Virol..

[6] Molinet A., Courtillon C., Bougeard S., Keita A., Grasland B., Eterradossi N., Soubies S. (2023). Infectious bursal disease virus: Predicting viral pathotype using machine learning models focused on early changes in total blood cell counts. Vet. Res..

[7] Rodríguez-Lecompte J.C., Niño-Fong R., Lopez A., Markham R.F., Kibenge F.S. (2005). Infectious bursal disease virus (IBDV) induces apoptosis in chicken B cells. Comp. Immunol. Microbiol. Infect. Dis..

[8] Yu Y., Li L., Sun R., Xu Z., Wang Q., Ou C., Zhang Y., Gao P., Ma J. (2021). Tissue distribution and developmental changes of PTEN in the immune organs of chicken and effect of IBDV infection on it. Poult. Sci..

[9] Alkie T.N. (2016). Rautenschlein S Infectious bursal disease virus in poultry: Current status and future prospects. Vet. Med. Res. Rep..

[10] Okata E.O., Al-Hassan R.M. (2023). Does publishing poultry vaccination schedule increase awareness and compliance among small-scale farmers? Evidence from Eastern Ghana. Cogent Food Agric..

[11] Arowolo O., George U., Luka P., Maurice N., Atuman Y., Shallmizhili J., Shittu I., Oluwayelu D. (2021). Infectious bursal disease in Nigeria: Continuous circulation of reassortant viruses. Trop. Anim. Health Prod..

[12] Badji A., Ducatez M., Lo F.T., Mbengue M., Diouf M., Samb Y., Diop M., Lo M.M., Thiongane Y., Guerin J.L. (2016). Genetic evolution of infectious bursal disease virus in Senegal. J. Vet. Med. Anim. Health.

[13] Omer M.G., Khalafalla A.I. (2022). Epidemiology and laboratory diagnosis of very virulent infectious bursal disease virus in vaccinated chickens in Khartoum, Sudan. Open Vet. J..

[14] Rukia S., Gabriel S., Joram B., Christopher K. (2020). Factors associated with infectious bursal disease vaccination failure in Dar es salaam, Tanzania. J. Vet. Med. Anim. Health.

[15] Gyening K., Corkish J. (1976). Infectious bursal disease in Ghana. Bull. Anim. Health Prod. Afr..

[16] Amakye-Anim J., Otsyina H., Osei-Somuah A., Aning K. (2008). Isolation and characterization of infectious bursal disease virus (IBDV) field strains and pathotypes in Ghana. Ghana J. Agric. Sci..

[17] Otsyina H., Osei-Somuah A., Amakye-Anim J., Aning K. (2009). An epidemiological study of recent outbreaks of Gumboro disease in Ghana. Ghana J. Agric. Sci..

[18] McFerran J., McNulty M., McKillop E., Connor T., McCracken R., Collins D., Allan G. (1980). Isolation and serological studies with infectious bursal disease viruses from fowl, turkeys and ducks: Demonstration of a second serotype. Avian Pathol..

[19] Eterradossi N., Saif Y.M. (2013). Infectious bursal disease. Diseases of Poultry.

[20] Fan L., Wu T., Hussain A., Gao Y., Zeng X., Wang Y., Gao L., Li K., Wang Y., Liu C. (2019). Novel variant strains of infectious bursal disease virus isolated in China. Vet. Microbiol..

[21] Mosad S.M., Elsayed M.M., Hammad E.M., Hendam B.M., Ali H.S., Eladl A.H., Saif M.A. (2024). Genotype classification and pathogenicity of infectious bursal disease virus circulating in vaccinated broiler chicken farms. Vet. Res. Commun..

[22] Michel L.O., Jackwood D.J. (2017). Classification of infectious bursal disease virus into genogroups. Arch. Virol..

[23] Jenberie S., Lynch S.E., Kebede F., Christley R.M., Gelaye E., Negussie H., Asmare K., Ayelet G. (2014). Genetic characterisation of infectious bursal disease virus isolates in Ethiopia. Acta Trop..

[24] Adel A., Zanaty A., Mosaad Z., Selim K., Hagag N.M., Badr M., Ellakany H., Shahien M., Samy A. (2024). Advancing IBDV diagnostics: A one-step multiplex real-time qRT-PCR for discriminating between vvIBDV and non-vvIBDV viruses, including the newly emerged IBDV variant. Front. Vet. Sci..

[25] Islam M.R., Nooruzzaman M., Rahman T., Mumu T.T., Rahman M.M., Chowdhury E.H., Eterradossi N., Müller H. (2021). A unified genotypic classification of infectious bursal disease virus based on both genome segments. Avian Pathol..

[26] Wang W., Huang Y., Zhang Y., Qiao Y., Deng Q., Chen R., Chen J., Huang T., Wei T., Mo M. (2022). The emerging naturally reassortant strain of IBDV (genotype A2dB3) having segment A from Chinese novel variant strain and segment B from HLJ 0504-like very virulent strain showed enhanced pathogenicity to three-yellow chickens. Transbound. Emerg. Dis..

[27] Islam M., Rahman S., Noor M., Chowdhury E., Müller H. (2012). Differentiation of infectious bursal disease virus (IBDV) genome segment B of very virulent and classical lineage by RT-PCR amplification and restriction enzyme analysis. Arch. Virol..

[28] Kasanga C., Yamaguchi T., Wambura P., Maeda-Machang’u A., Ohya K., Fukushi H. (2007). Molecular characterization of infectious bursal disease virus (IBDV): Diversity of very virulent IBDV in Tanzania. Arch. Virol..

[29] Jiang N., Wang Y., Zhang W., Niu X., Huang M., Gao Y., Liu A., Gao L., Li K., Pan Q. (2021). Genotyping and molecular characterization of infectious bursal disease virus identified in important poultry-raising areas of China during 2019 and 2020. Front. Vet. Sci..

[30] Jackwood D.J., Schat K.A., Michel L.O., de Wit S. (2018). A proposed nomenclature for infectious bursal disease virus isolates. Avian Pathol..

[31] Letunic I., Bork P. (2019). Interactive Tree Of Life (iTOL) v4: Recent updates and new developments. Nucleic Acids Res..

[32] Pikuła A., Domańska-Blicharz K., Cepulis R., Śmietanka K. (2017). Identification of infectious bursal disease virus with atypical VP2 amino acid profile in Latvia. J. Vet. Res..

[33] Brown M.D., Green P., Skinner M.A. (1994). VP2 sequences of recent European ‘very virulent’isolates of infectious bursal disease virus are closely related to each other but are distinct from those of ‘classical’strains. J. Gen. Virol..

[34] Nwagbo I.O., Shittu I., Nwosuh C.I., Ezeifeka G.O., Odibo F.J., Michel L.O., Jackwood D.J. (2016). Molecular characterization of field infectious bursal disease virus isolates from Nigeria. Vet. World.

[35] Jackwood D.J., Sommer-Wagner S. (2007). Genetic characteristics of infectious bursal disease viruses from four continents. Virology.

[36] Beshah A., Ahmed A., Dandecha M. (2024). Epidemiology And Risk Factors of Infectious Bursal Disease-A Review. Int. J. Med. Case Rep. Rev..

[37] Rashid M.H., Xue C., Islam M.T., Islam M.R., Cao Y. (2013). Risk factors associated with infectious bursal disease in commercial chickens in Bangladesh. Prev. Vet. Med..

[38] Mutinda W.U., Nyaga P.N., Mbuthia P.G., Bebora L.C., Muchemi G. (2014). Risk factors associated with infectious bursal disease vaccination failures in broiler farms in Kenya. Trop. Anim. Health Prod..

[39] Okoye J., Uzoukwu M. (1981). An outbreak of infectious bursal disease among chickens between 16 and 20 weeks old. Avian Dis..

[40] Mohammed U.M., Garba S., Armiya’u R. (2019). Outbreak of infectious bursal disease in a flock of 14 weeks old ISA brown pullets, Sokoto State, Nigeria. GSC Biol. Pharm. Sci..

[41] Bumstead N., Reece R., Cook J.K. (1993). Genetic differences in susceptibility of chicken lines to infection with infectious bursal disease virus. Poult. Sci..

[42] Tippenhauer M., Heller D.E., Weigend S., Rautenschlein S. (2013). The host genotype influences infectious bursal disease virus pathogenesis in chickens by modulation of T cells responses and cytokine gene expression. Dev. Comp. Immunol..

[43] Bowan P.A. (2022). Assessment of Drinking Tap Water Quality in Wa Municipality, Ghana. J. Environ. Sci. Stud..

[44] Guérin J.-L., Balloy D., Pinson M., Jbenyeni A., Delpont M. (2024). Vaccination technology in poultry: Principles of vaccine administration. Avian Dis..

[45] Orakpoghenor O., Oladele S.B., Abdu P.A. (2020). Research Note: Detection of infectious bursal disease virus antibodies in free-living wild birds in Zaria, Nigeria. Poult. Sci..

[46] YOngshan W., Zongan Z., Yv L., Jibo H., Kongwang H., Chan D., Xiaozhao D., Jian G., Zhenyv D. (2003). Isolation of infectious bursal disease virus from different bird species and analysis of their biological properties. Dongwu Yixue Jinzhan.

[47] Jeon W.-J., Lee E.-K., Joh S.-J., Kwon J.H., Yang C.-B., Yoon Y.-S., Choi K.-S. (2008). Very virulent infectious bursal disease virus isolated from wild birds in Korea: Epidemiological implications. Virus Res..

[48] Naggar R.F.E., Rohaim M.A., Munir M. (2020). Potential reverse spillover of infectious bursal disease virus at the interface of commercial poultry and wild birds. Virus Genes.

[49] Wagari A. (2021). A review on infectious bursal disease in poultry. Health Econ. Outcome Res. Open Access.

[50] Aliyu H., Sa’idu L., Jamilu A., Andamin A. (2016). Akpavie S Outbreaks of virulent infectious bursal disease in flocks of battery cage brooding system of commercial chickens. J. Vet. Med..

[51] Jenbreie S., Ayelet G., Gelaye E., Kebede F., Lynch S.E., Negussie H. (2012). Infectious bursal disease: Seroprevalence and associated risk factors in major poultry rearing areas of Ethiopia. Trop. Anim. Health Prod..

[52] Zhang W., Wang X., Gao Y., Qi X. (2022). The Over-40-years-epidemic of infectious bursal disease virus in China. Viruses.

[53] Gul I., Hassan A., Muneeb J.M., Akram T., Haq E., Shah R.A., Ganai N.A., Ahmad S.M., Chikan N.A., Shabir N. (2023). A multiepitope vaccine candidate against infectious bursal disease virus using immunoinformatics-based reverse vaccinology approach. Front. Vet. Sci..

[54] Müller H., Mundt E., Eterradossi N., Islam M.R. (2012). Current status of vaccines against infectious bursal disease. Avian Pathol..

[55] Baba S. (2006). Avian influenza and family poultry in Nigeria: Potentials for rapid spread and continued presence of disease. World’s Poult. Sci. J..

[56] Goka P.D., Folitse R.D., Tasiame W., Amemor E., Burimuah V., Asare D.A., Emikpe B.O. (2024). Assessing cross-border live poultry trade as a possible factor for infectious diseases spread between Aflao and Lomé. PAMJ-One Health.

[57] Pikuła A., Lisowska A., Jasik A., Perez L.J. (2021). The novel genetic background of infectious bursal disease virus strains emerging from the action of positive selection. Viruses.

